# A neuromorphic event data interpretation approach with hardware reservoir

**DOI:** 10.3389/fnins.2024.1467935

**Published:** 2024-11-14

**Authors:** Hanrui Li, Dayanand Kumar, Nazek El-Atab

**Affiliations:** SAMA Labs, Computer, Electrical and Mathematical Science and Engineering Division, King Abdullah University of Science and Technology (KAUST), Thuwal, Saudi Arabia

**Keywords:** memristor, reservoir computing, event representation, SNN, neuromorphic computing

## Abstract

Event cameras have shown unprecedented success in various computer vision applications due to their unique ability to capture dynamic scenes with high temporal resolution and low latency. However, many existing approaches for event data representation are typically algorithm-based, limiting their utilization and hardware deployment. This study explores a hardware event representation approach for event data utilizing a reservoir encoder implemented with analog memristor. The inherent stochastic and non-linear characteristics of the memristors enable the effective and low-cost feature extraction of temporal information from event streams as a reservoir encoder. We propose a simplified memristor model and memristor-based reservoir circuit specifically for processing dynamic visual information and extracting feature in event data. Experimental results with four event datasets demonstrate that our approach achieves superior accuracy over other methods, highlighting the potential of memristor-based event processing system.

## 1 Introduction

Event cameras are bio-inspired vision sensors that operate differently from conventional frame-based sensors. They are sensitive to detect the brightness change based on scene dynamics rather than capturing image's frame at a fixed rate for frame-based camera (Gallego et al., 2020). The event behavior in individual pixel is triggered only when the brightness intensity change exceeds the threshold without waiting for the command of the global shutter (Furmonas et al., 2022). Unlike the conventional camera that outputs an analog value in each pixel, the dynamic vision camera (DVS) produces a serials of events or spikes, which weigh only 1 bit (Lichtsteiner et al., 2008). The unique properties of event camera offer high temporal resolution and high dynamic range with better energy efficiency and latency compared with traditional cameras. With faster motion of objects, more events are generated as each pixel adjusts the delta modulator sampling rate according to the change of the logarithm of optical intensity. This makes event cameras exceptionally fast and efficient for edge applications, such as surveillance or monitoring, where only motion or change relevant (Delbruck and Lang, 2013; Glover and Bartolozzi, 2016).

However, traditional computer vision algorithms cannot be directly used to process event data as it only contains binary information of asynchronous brightness intensity change. How to effectively process event data still remains an issue. These event data are asynchronous spike behavior with microsecond-level resolution, which requires special algorithm or specialized hardware for good representation or prepossessing. Many studies have been proposed to realize good event data representation. Gehrig et al. (2019) proposed differentiable operation method to convert event streams into grid-based representations. Sironi et al. (2018) introduced a local memory sharing method with time surface, which converts event stream into an image with function of the motion history at that location. The other common method is to integrate event behaviors to frames by slicing a constant time window or fixed number of event packets (Everding and Conradt, 2018). Nevertheless, these methods are mainly based on the software level optimization without the implementation of hardware.

The memristor is an emerging non-volatile device, which shows the dynamic conductance range with programming ability (Khalid, 2019). It is a two terminal device, which retains a state of resistance with flexible response to varying electrical inputs. Many memristor devices exhibit the short-term-memory (STM) behavior, which has the non-linearity and memory decay characteristics in response to a stimulus as optical or electrical input (Kumar et al., 2023; Hu et al., 2021; Wu et al., 2023; Jo et al., 2010). It allows the devices to map temporal input patterns into collective memristor resistance states, which can be viewed as a dynamic reservoir node. Many research studies have been proposed to implement hardware reservoir computing based on memristor (Zhong et al., 2021, 2022; Sun et al., 2021). Such properties can efficiently process event data and extract information through the natural behavior of the device. The memristor reservoir encoder takes advantage of both spike neural network (SNN) and reservoir computing, which is effective to capture features in high dimensional space and resembles the processes of visual cognition (Chen et al., 2024). This approach provides a solution for event representation at a low cost, which is suitable for edge applications including robotic systems, real-time monitoring, and complex signal processing.

In this study, we propose a neuromorphic approach for event-based data interpretation (NEIR), which utilizes memristor-based circuit as reservoir encoder to encode event data. The stochastic nature and non-linear property enables the reservoir layer to effectively extract the event-based information at a low cost. We also propose a simplified memristor model based on the VTEAM model with time-surface behavior to fit the non-linear property. The state of memristor in reservoir circuit changes according to the input of event streams, and the result of extracted information is represented by the generated current. The reservoir layer allows the effective feature extraction for the temporal information in all prior spike inputs produced by the event camera without any use of dedicated memory units and logic circuits for complex preprocessing algorithms. Our design is triggered by the digital signal with ON and OFF events, which eliminates the need for Digital-to-Analog Converter (DAC) requirements. It is similar as retina cells that can directly sense and encode raw, asynchronous visual inputs at low cost. This scheme introduces a novel approach for event-data encoding with memristor-based reservoir node, which highlights the potential application in this field.

The contributions of this study are as follows:

1. We introduce a hardware system called NEIR which utilizes a memristor-based circuit to encode event data. The memristor array can effectively simulate the reservoir state as hardware implementation with non-linear property.
2. We propose a simplified memristor model specifically designed for the pulse behavior, which is suitable for non-linear behavior modeling and event-based data representation. It is an intuitive and easy-to-use framework derived by the VTEAM model to capture the pulse stimulus with less parameters.
3. We compare our memristor encoding method with other event representation methods with four event-based datasets. Our evaluations demonstrate that our encoding method can achieve the highest accuracy across the same bottleneck structure on both spike and non-spike models.

The remainder of this study is organized as follows. We introduce in Section 2 previous studies in both reservoir computing and memristor areas. In Section 3, we provide a detailed description of our proposed method. The performance of this approach is evaluated in Section 4. We then provide a discussion on experimental results and performance in Section 5. Finally, the study is concluded in Section 6.

### 1.1 Related work

Reservoir computing is a widely used machine learning framework known for its non-linear and dynamic characteristics (Yan et al., 2024). This approach facilitates the handling of complex temporal patterns without the need for extensive retraining or parameter adjustments inherent in traditional models (Cucchi et al., 2022). By leveraging a fixed number of randomly generated neurons in the reservoir to extract information in high dimensions, it allows for efficient computation and robust performance across a variety of temporal-spatial tasks (Tanaka et al., 2019). For hardware implementation, the memristor is a popular candidate to work as the physical reservoir due to its non-linear and dynamic programming properties (Cao et al., 2022). The memristor has shown its unique short-term or long-term memory with both optical and electrical programming abilities. It can work as the reservoir nodes to perform non-linear information transformation (encoding) of the temporal input data into the stored reservoir states (Zhang et al., 2023). In such systems, the reservoir node encodes the spatiotemporal information naturally by device dynamics, which eliminates the need for external memory or arithmetic and logic units (ALUs). By implementing physical reservoir with memristors, it can achieve outstanding energy efficiency and power consumption as the number of nodes is fixed.

The STM characteristic of the memristor brings its non-linear and time-dependent properties, which can serve as a suitable hardware platform for the dynamics required for reservoir computing. Many studies have shown the potential ability to utilize memristor-based circuit as the reservoir node and take advantage of the storage ability (Liang et al., 2024; Yan et al., 2024; Yang et al., 2024). Wu et al. (2023) integrated organic light-sensing materials into memristors to simulate an optical reservoir system with a spike neuromorphic network for pattern recognition. Yang et al. (2024) proposed a mask reservoir computing circuit using memristor arrays to process analog biomedical signals. Zhou et al. (2023) proposed light-responsive vision sensors that convert dynamic motion into event signals and extract features for efficient motion recognition. These studies have integrated memristors into reservoir computing platforms, offering potential opportunities for hardware-based reservoir computing applications.

However, most of the existing studies primarily concentrate on device characteristics and software simulations. In this study, we provide a general event representation method via memristor modeling and circuit design for reservoir computing, which enhances the event-based feature extraction with low energy consumption.

## 2 Memristor model and reservoir computing

**Figure 1:**
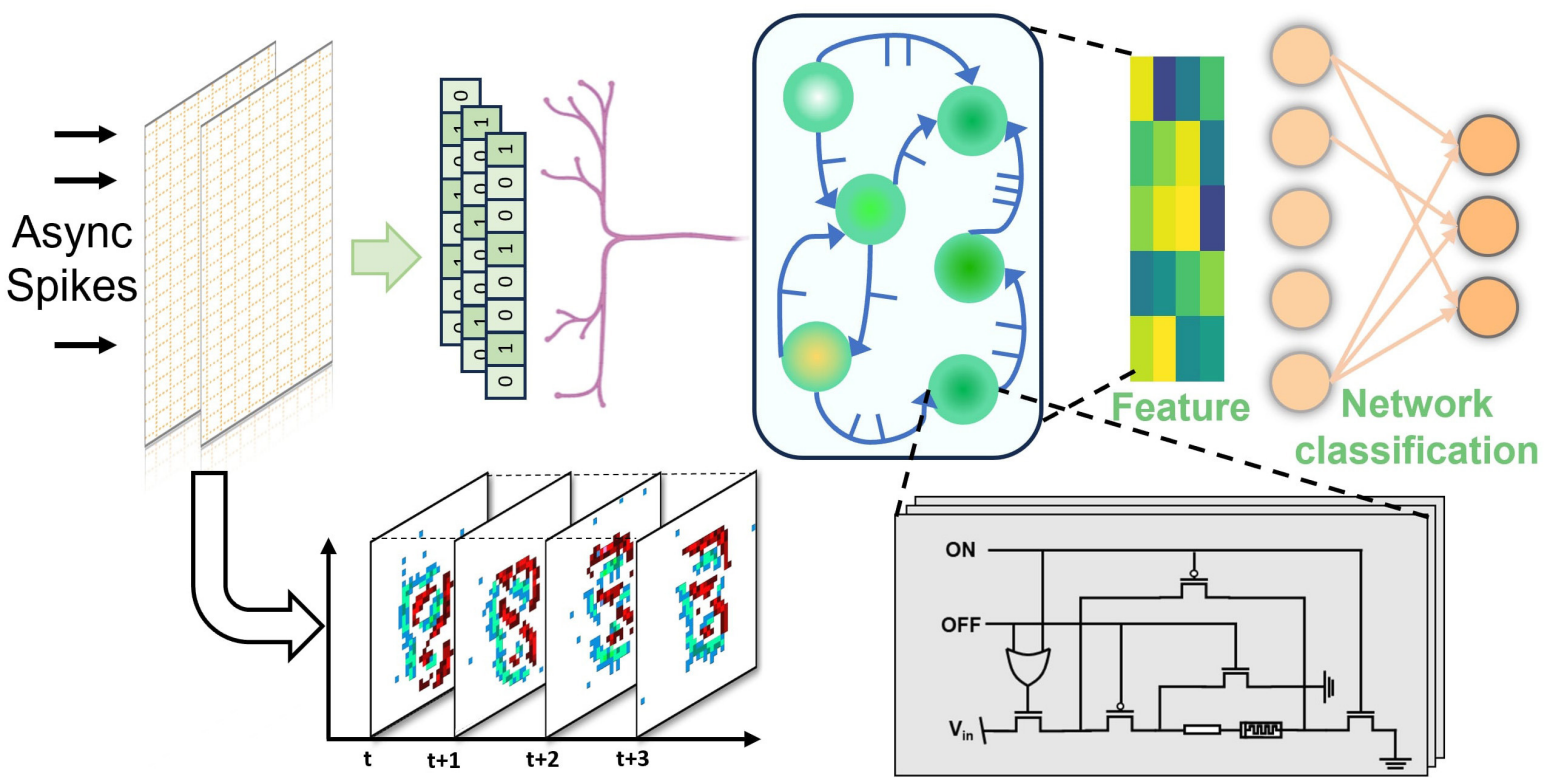
NEIR structure: asynchronous spike inputs are directly sent to hardware reservoir layer to extract the feature. The neural network can be implemented for further classification.

Figure 1 shows the reservoir computing system implemented for event data processing. For event data processing, the memristor-based reservoir layer has the non-linear dynamic property to respond to stimuli, where both optical and electrical pulse can be viewed as potential stimuli (Pereira et al., 2023). The asynchronous events generated by event camera can be directly sensed and processed by the reservoir layer without any configuration of external logic circuits. The bipolar terminal in memristor enables the flexibility and reconfigurability of programming to different conductance values with effective feature extraction ability. The STM property of the memristor device offers the superior ability to react with temporal information and decay with time surface, which could work as the encoder for event data to extract information (Moon et al., 2019). The feature map extracted by the reservoir layer is sent to a classification bottleneck network for identification. The network including convolutional layer and fully connected layer can be easily implemented by memristor crossbar with high energy efficiency and low power consumption.

### 2.1 Memristor-based circuit unit

**Figure 2:**
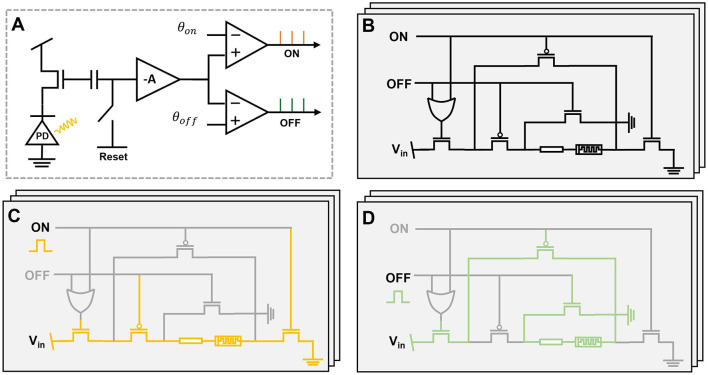
**(A)** The circuit diagram of DVS sensor. **(B)** The circuit diagram of memristor-based reservoir node. **(C)** The circuit diagram when ON signal is activated. **(D)** The circuit diagram when OFF signal is activated.

The memristor circuit unit plays as the basic element of reservoir node for event data interpretation. It is used to extract high dimension information and behave as non-linear state of reservoir. The DVS camera measures output with event behavior through the brightness change (Lichtsteiner et al., 2008). The pixel circuit is shown in Figure 2A, where the brightness change over threshold value of comparator is generated as ON and OFF event. The asynchronous event behaviors can be directly fed and processed by the proposed reservoir circuit as shown in Figure 2B. The ON and OFF signal represents the digital control signal of event data, while the *V*_*in*_ is the constant voltage supply for memristor programming. By definition of event data, the ON and OFF signal cannot be activated simultaneously as brightness change can be either positive or negative. The schematic diagram is shown in Figures 2C, D when ON and OFF signal is activated at a high level. The event data can drive the switch control of voltage, where the memristor state changes accordingly with the event type and stores the previous integrated information. The bipolar programming of the memristor device provides the flexibility of reservoir state modification with non-linear dynamics. According to the memristor readout, the multiplexer with clock control is utilized to read the conductance of each memristor simultaneously. During the read phase, a small voltage (< 0.15 V) is applied across the memristor to prevent any alteration of its conductance state. A transimpedance amplifier (TIA) can be utilized to convert the small input current into a proportional voltage signal for further processing.

### 2.2 A simplified memristor model

The VTEAM model is a popular voltage-controlled model considering the threshold voltage phenomenon, which is compatible with many window functions (Kvatinsky et al., 2015). In addition, the VTEAM model has great flexibility to simulate the non-linear dopant drift phenomenon. Considering the STM behavior in many solid-state memory devices, we improve the memristor model by adding a decay term below the threshold value. The optimized VTEAM model is shown below:


(1) $$\frac{dw(t)}{dt}=\{\begin{array}{ll} k_{\text{off}}\cdot {\left(\frac{v(t)}{v_{\text{off}}}-1\right)}^{\alpha_{\text{off}}}\cdot f(x(t)), & 0<v_{\text{off}}<v\\ -\frac{w(t-1)}{\tau }, & v_{\text{on}}<v<v_{\text{off}}\\ k_{\text{on}}\cdot {\left(\frac{v(t)}{v_{\text{on}}}-1\right)}^{\alpha_{\text{on}}}\cdot f(x(t)), & v<v_{\text{on}}<0 \end{array}$$

(2) $$\begin{array}{l} x\left(t\right)=\frac{w\left(t\right)}{W} \end{array}$$

(3) $$\begin{array}{l} R\left(t\right)=R_{\text{on}}+\left(R_{\text{off}}-R_{\text{on}}\right)\cdot x\left(t\right) \end{array}$$

(4) $$\begin{array}{l} v\left(t\right)=R\left(t\right)\cdot i\left(t\right) \end{array}$$

where *w*(*t*) is an internal state variable in [0, W], *W* is a constant that represents the maximum value of *w*, *x*(*t*) is an internal state variable in [0, 1], τ is the constant decay value, *f*(*x*) is the window function, *v*(*t*) is the voltage applied to the memristor, *i*(*t*) is the current passing through the memristor, *R*(*t*) is the resistance of the memristor, and *t* is the time. The parameters *v*_*on*_ and *v*_*off*_ are threshold voltages, and *R*_*on*_ and *R*_*off*_ are resistance values of the memristor in its ON and OFF states. The parameters *k*_*on*_, *k*_*off*_, α_*on*_, and α_*off*_ are constants. The window function *f*(*x*) is applied to model the non-linear drift behavior with scalable parameters (Soni and Sahoo, 2022).

Considering the dynamic behavior in the time surface, the state change with the memristor can be modeled as follows:


(5) $$\begin{array}{l} △R=\left(R_{off}-R_{on}\right)\cdot △x=\left(R_{off}-R_{on}\right)\cdot \frac{△w}{W} \end{array}$$

In the VTEAM model, the memristor's resistance change is primarily governed by the direction and magnitude of the voltage. When the memristor device is applied under serial constant voltage pulses δ_*spk*_(*t*) over than threshold *v*_on_ and *v*_off_, the internal state variable *w* behaves as below:


(6) $$\begin{array}{ll} △w & =\{\begin{array}{ll} -c_{1}\cdot f(x), & \delta_{spk}(t)=1\\ c_{2}\cdot f(x), & \delta_{spk}(t)=-1 \end{array} \end{array}$$

where *c*_1_ and *c*_2_ denote constants defining how much the resistance changes within the time according to pulse state. The state change △*R* is dependent on internal state *w* and can be expressed as below:


(7) $$\begin{array}{l} \frac{dR\left(t\right)}{dt}=\frac{R\left(t-1\right)}{\tau }\cdot 1_{\delta_{\left(t\right)}=0}+c\cdot \delta_{spk}\left(t\right)\cdot f\left(x\right) \end{array}$$

where *1* is the indicator function that equals 1 if δ_*spk*_(*t*) = 0 and takes the value 0 in other cases, and *f*(*x*) is the appeid window function. In this case, we consider the boundary effect and apply Biolek's window function (Biolek et al., 2009), which is given by *f*(*x*) = 1−(*x*−*stp*(−*i*))^2*p*^, where *stp* is the step function. We substitute the parameter *x* with Equation 3, and the window function can be expressed as follows:


(8) $$\begin{array}{l} f\left(x\right)=1-{\left(\frac{R-R_{on}}{R_{off}-R_{on}}-stp\left(-i\right)\right)}^{2p} \end{array}$$

Our model in NEIR method is derived from the VTEAM model yet it is significantly simplified. Compared with the other existing memristor model, our model focuses exclusively on pulse behavior instead of I-V behavior. It has the much simplified model with less fitting parameters (4 in total), which is easy to use in pulse simulation. This approach relies on the memristor's response under the pulse stimulus, which is a more intuitive framework that prioritizes efficiency in electrical pulse manipulation. This direct and effective method provides a particular advantage for simulating the dynamic memristor property for reservoir computing scenarios, where time-dependent spike data can be treated as pulse stimulus. By mirroring the natural processing mechanisms in biological neurons, this method allows for physical implementation in memristors responding to electrical or optical impulses over time.

### 2.3 Behavior simulation

**Figure 3:**
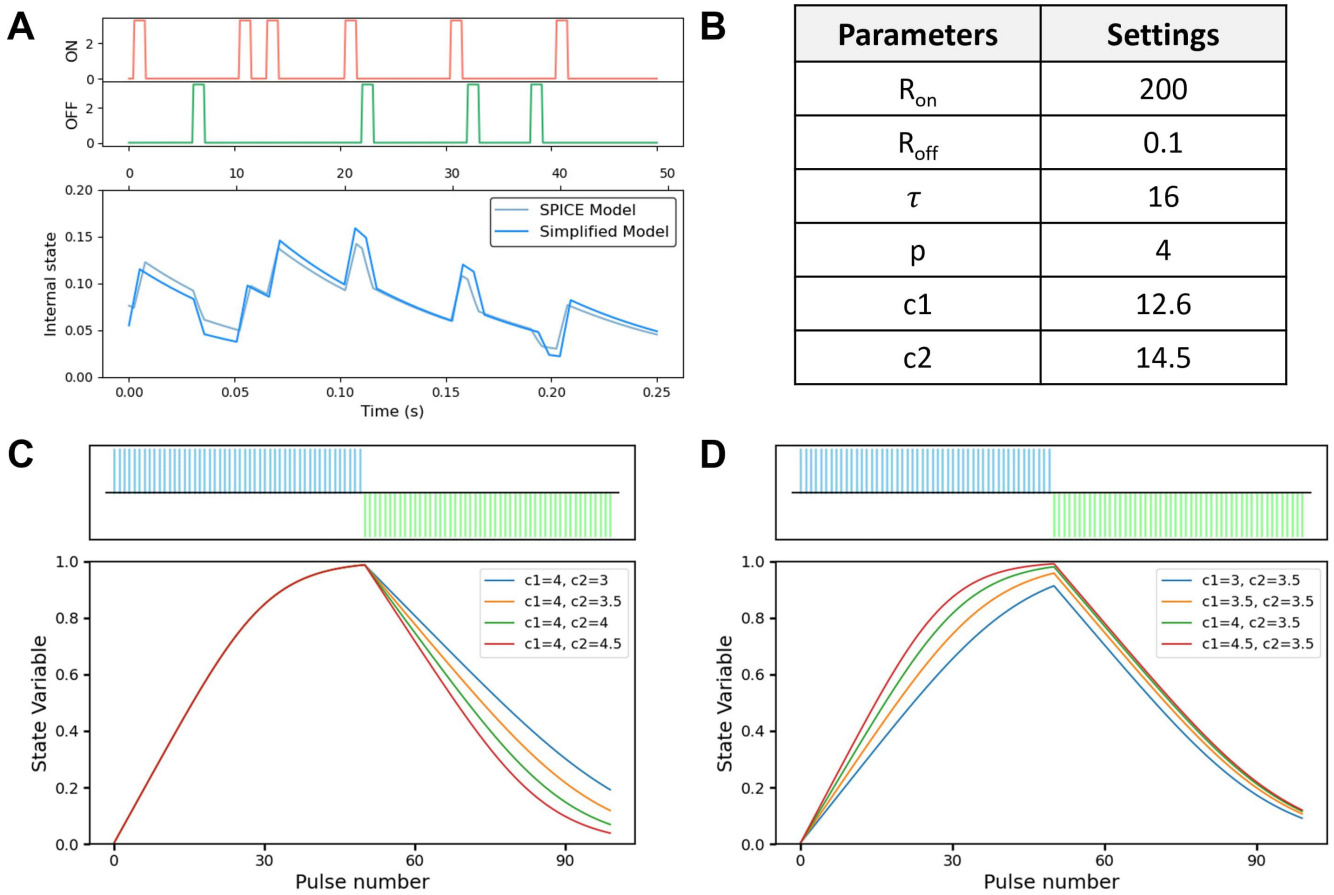
**(A)** Simulation result comparison between SPICE model and our simplified model. **(B)** The list of parameters for simulation. **(C, D)** Proposed simplified memristor model with variable parameter *c*1 and *c*2.

To demonstrate the effectiveness and precision of modeling the memristor device property, we conducted a comparative simulation using serials of random pulses with SPICE-based model and our simplified model as shown in Figure 3A. Based on the proposed reservoir node circuit, the SPICE simulation is conducted with the modified VTEAM model. The simplified model aims to capture the pulse behavior of the memristor state with simulation frequency of 200 Hz. For demonstration purposes, we increase the event frequency and decay penalty, whereas in real scenarios, events usually fire at low rates with sparsity. The well-fitting result indicates that our simplified model can accurately extract spike-based features and retain critical dynamics, which makes it a valuable tool for fast and efficient modeling. The parameters of the simplified memristor behavior simulation in this study are listed in Figure 3B. From Equation 7, it shows the strong non-linear relationship between programming pulses and the resistance value of the memristor, which is essential for the reservoir computing simulation due to the non-linearity and dynamics. The simulated memristor model for a pair of positive and negative pulses is shown in Figures 3C, D. The resistance change curve shows a non-linear manner, where state variable *x* gradually increases with positive pulses and decreases with negative pulses. The resistance is limited between *R*_*on*_ and *R*_*off*_ with boundary effect constraint. The non-linearity value of the curve can be easily modified by two parameters c1 and c2 or through curve fitting with experimental memristor data. The model is simplified and purely based on the pulse mode with few parameters, which is easy to use in non-linear fitting cases.

The event behavior can be described and transmitted with three essential parameters, including time step, pixel coordinates (x, y position), and event polarity (Leñero-Bardallo et al., 2018). The event behavior can be expressed as


(9) $$\begin{array}{l} e_{i}=\left\{x_{i},y_{i},p_{i},t_{i}\right\} \end{array}$$

where *x*_*i*_ and *y*_*i*_ are the positions of an active pixel, *p*_*i*_ represents the polarity of an event, *t*_*i*_ is the time step, and *e*_*i*_ is the ith event in the stream. For the reservoir encoding method, the memristor array updates its conductance state according to the event streams. We partition the event recordings into smaller chunks and perform feature extraction by reading the current periodically at each segment. These patterns in an analog state can be processed by subsequent DNN/SNN blocks. The proposed encoding method is shown in Algorithm 1.

**Algorithm 1:**
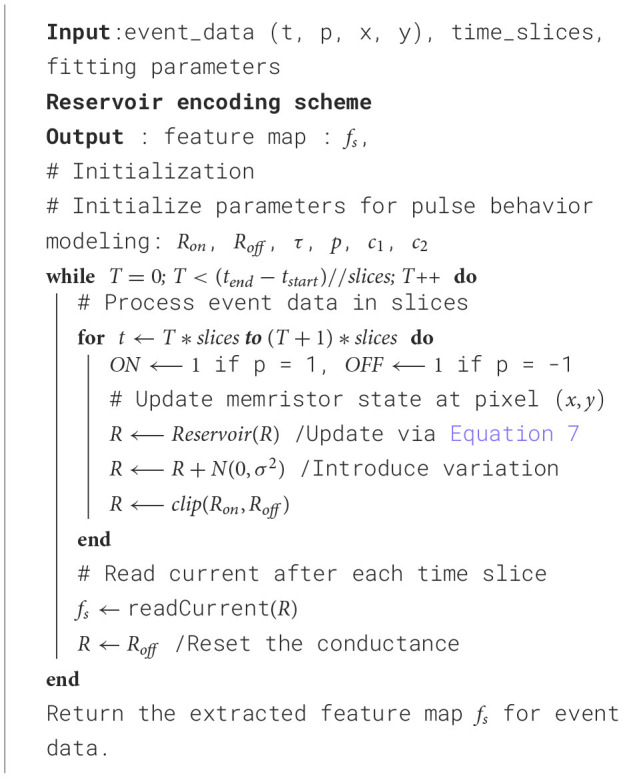
The proposed encoding method.

### 2.4 Event-data representation methods and result visualization

Event data are processed and transformed into alternative representations to extract significant information and facilitate specified algorithm processing. These representation methods focus on converting raw event data into a format that is more amenable to algorithmic processing, enabling efficient information extraction and decision-making (Lakshmi et al., 2019). Our reservoir layer serves as the encoder with the same purpose but applying a memristor-based hardware framework with non-linear dynamics. This is distinct from traditional methods and aligns with our objective to optimize both performance and hardware compatibility. In this study, we make a comparison of existing popular representation methods for event data in Table 1. This comparison presents the characteristics and storage feature of each method, which highlight their suitability for different computational scenarios. Our proposed memristor-based method can extract both spatial and temporal features with the advantage of being hardware-friendly and energy-efficient.

**Table 1:** Comparison of event representation methods.

**Event representation method**	**Features**	**Algorithm complexity**	**Properties**
Individual event	Spatial	Low	Sparse/High data rate
Event packet	Spatial	Low	Simple/Low precision
Time surface	Spatial and temporal	High	Decay over time/Temporal information
VoxelGrid	Temporal better than spatial	Moderate	Avoid event collapse/High precision
Ours	Spatial and temporal	High but hardware-friendly	Energy-efficient/High precision

**Figure 4:**
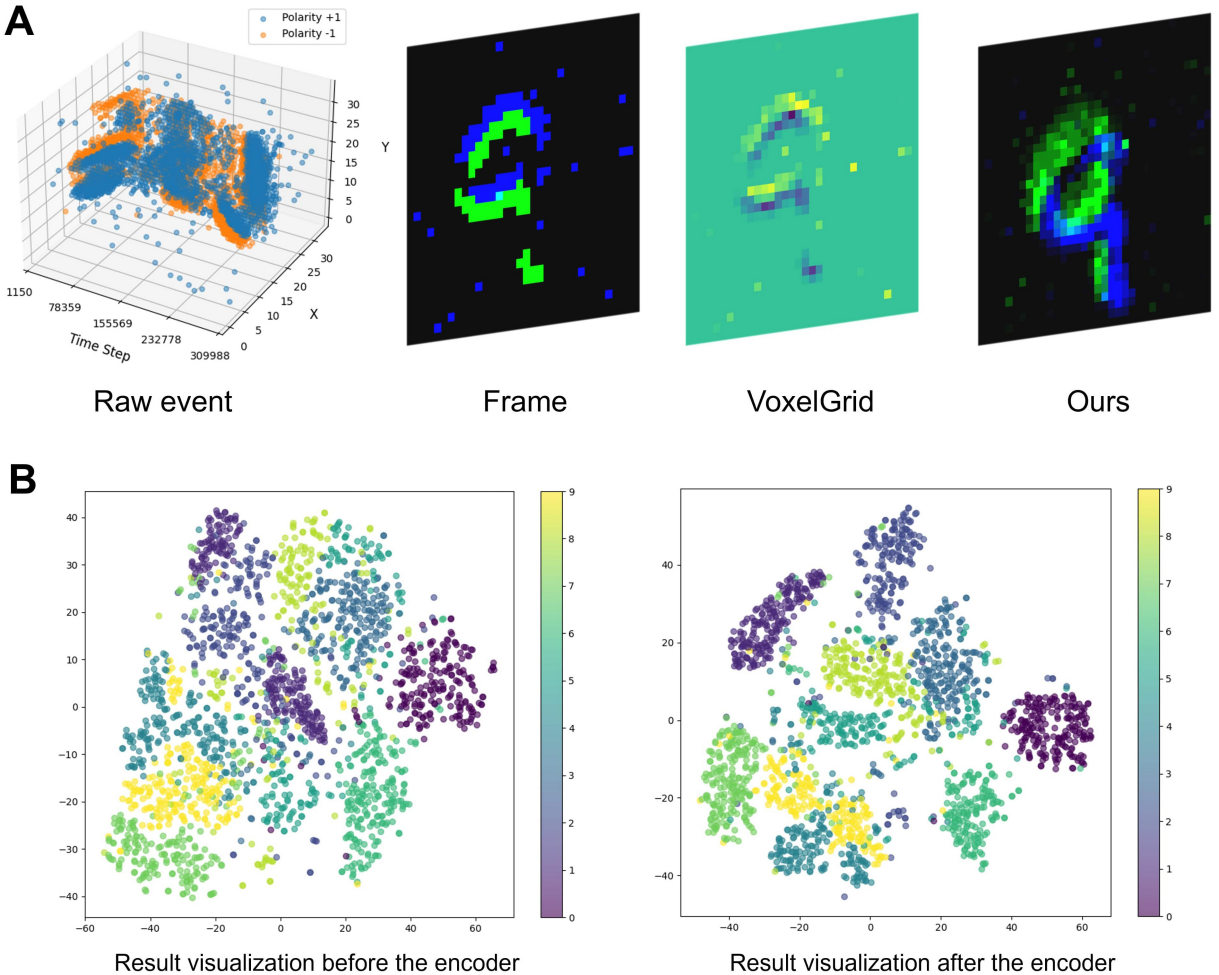
**(A)** Different event representation result visualization: Raw event behavior, Integrate-to-frame, VoxelGrid, and our method. **(B)** T-SNE visualization of NMNIST dataset before and after the proposed encoder.

Figure 4A displays the visualization result of different event representation methods for digit “9” in NMNIST dataset. The visualization result contains four different methods, namely, raw event, frame, VoxelGrid, and our method. The raw event data consist of individual events plotted in space and time, resulting in a sparse and scattered visualization. This method retains all event information but lacks structure, making it difficult to discern the underlying pattern of the digit “9.” The frame-based representation method accumulates events over a fixed time interval to create a frame, which could blur temporal information and introduce motion artifacts. This VoxelGrid approach balances both spatial and temporal information but may suffer from quantization effects, which may lead to a loss of fine details. Our method extracts both spatial and temporal information through non-linear behavior of memristor device, maintaining clarity in the contours. The event representation methods aim to capture and retain essential features through sparse event behaviors, enabling efficient processing and analysis of dynamic visual scenes. Figure 4B shows the comparison result for raw event data and the data after memritsor-based reservoir encoder. The visualization result utilizes t-distributed stochastic neighbor embedding (t-SNE) method for dimensionality reduction. It contains 2,000 samples randomly chosen from test set of NMNIST dataset with the same experimental setup. Samples from the same category are more distinctly clustered after the proposed encoder, which indicates the information extraction ability of proposed memristor reservoir event representation method.

## 3 Numericial experiment

### 3.1 Experiments on DVS dataset

We evaluate the memristor encoding method in DNN/SNN bottleneck structure through event-based datasets, including DVS Gesture (Amir et al., 2017), DVS CIFAR10 (Li et al., 2017), NMNIST (Orchard et al., 2015), and DVSLip (Tan et al., 2022). To integrate the information effectively, the encoder captures data at a frequency of 30 Hz and sent to the subsequent layers. In the simulation, the initial states of neurons in the reservoir are drawn from a normal distribution with a mean of (Rmin+Rmax)/2. The NEIR method allows efficient feature description of the temporal information in all prior spike inputs with simple DNN backpropagation rules across time steps. We compare the NEIR with existing encoding approaches including frame, time surface, and voxel grid. The comparison results are shown in Table 2. With the same experiment setting, we evaluate the event representation methods via spike and non-spike bottleneck network. For image recognition on DVS Gesture dataset, our NEIR method reaches the accuracy of 88.42% in VGG 11 model, which is much higher than the other encoding method (87.11% for time surface).

**Table 2:** Performance comparison of different Encoding methods.

**Dataset**	**Encoding method**	**Bottleneck model**	**Accuracy (DNN)**	**Accuracy (SNN)**
DVSGesture	Frame	VGG 11	77.27	72.43
	Time surface	VGG 11	87.11	69.30
	VoxelGrid	VGG 11	80.34	71.96
	Ours	VGG 11	88.42	74.18
DVSCIFAR10	Frame	VGG 16	65.12	61.30
	Time surface	VGG 16	68.42	64.95
	VoxelGrid	VGG 16	66.10	60.78
	Ours	VGG 16	72.45	66.45
NMNIST	Frame	Three-layer ANN	89.48	86.92
	Time surface	Three-layer ANN	96.76	95.85
	VoxelGrid	Three-layer ANN	97.48	94.84
	Ours	Three-layer ANN	98.15	97.45
DVSLip	Frame	ResNet-18	38.54	33.97
	Time surface	ResNet-18	47.54	41.74
	VoxelGrid	ResNet-18	50.89	38.13
	Ours	ResNet-18	53.13	44.81

We also apply spike-based bottleneck network to evaluate the performance of encoding methods. To solve the non-differentiability of spike behavior, we implement the spiking activation function with an approximation of gradient (Wu et al., 2019). We set the default time steps as 5 for each event sample and conduct experiment with different encoding methods. We observed that our method achieved an accuracy of 66.45% on the DVS CIFAR10 dataset using a spike-based VGG 16 model, compared with 64.95% when employing time-surface techniques. The NEIR method exhibits superior performance over other encoding method in both spike and non-spike architecture. The ablation study demonstrates that the NEIR method achieves high accuracy on the test dataset, indicating its effectiveness in capturing essential features from event data. The Pytorch platform and Tonic package were used for all the experiments with methods described above.

### 3.2 Effects of device variation and ADC resolutions

**Figure 5:**
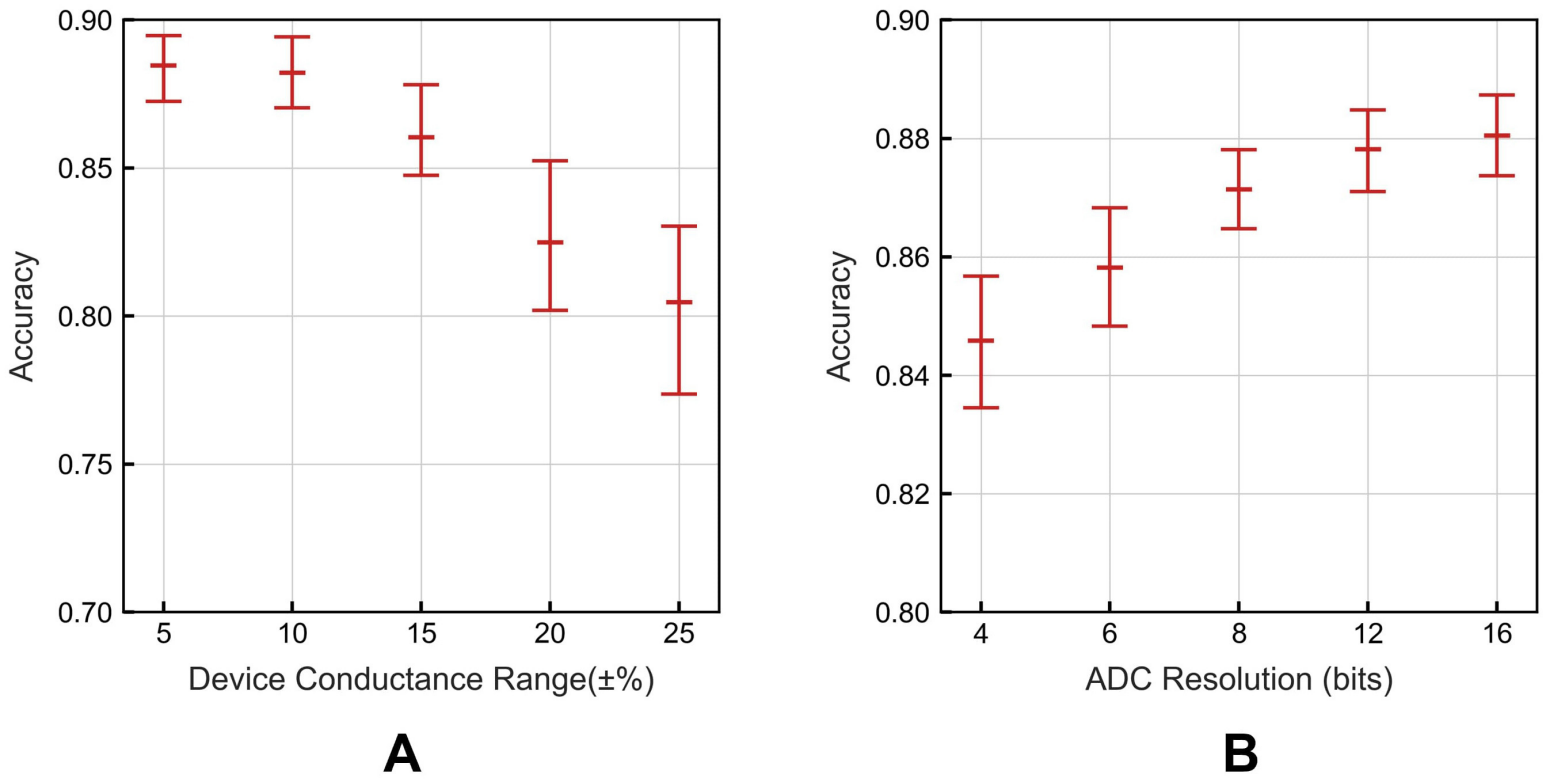
**(A)** The effect of device conductance variation. **(B)** The effect of ADC resolutions.

Figures 5A, B provide a summary of the impact of non-idealities on our system for image recognition on DVS Gesture dataset. Each data point in the figure shows the mean and standard deviation across five arbitrary seed values. Due to the stochastic ion behavior and vacancy forming process, the conductance of memristors displays variations and fluctuations from expected conductance value (Ielmini and Wong, 2018). Figure 5A illustrates the impact of device conductance variation. As device conductance variation increases, network performance gradually declines, dropping from 88.45% at a 5% conductance range to 80.47% at a 25% conductance range, with an increasing of accuracy variation. Figure 5B shows the effect of ADC resolution. With the higher resolution ADC, the precision of information improves and the network achieves better performance.

### 3.3 Performance evaluation

Table 3 shows the hardware evaluation and comparison for the proposed reservoir circuit node. We conduct the circuit simulation via PSPICE in Cadence and explore critical performance metrics including power consumption, energy usage, and latency. The result shows an average power consumption of 137 μW and normalized energy of 0.69 μJ per event with supply voltage of 3.3V, which highlights efficiency in information encoding at low cost. The expected propagation delay of 280.19 ns represents the total time delay across the critical path, where the memristor crossbar analysis is evaluated in Lu et al. (2021). Compared to other studies, our design demonstrates competitive performance. Zhong et al. (2021) reports a lower power consumption of 50 μW and a normalized energy of 0.006 μJ but emphasized on device measurement. Our evaluation is based on the individual memristor reservoir circuit and achieve competitive accuracies on multiple event-based datasets, which is more complex task across others. This circuit performance evaluation shows the requirements for the memristor-based reservoir node, which reveals the potential application for energy-efficient event sensing system.

**Table 3:** Performance comparison of proposed reservoir node.

**References**	**Power (μW)**	**Normalized energy (μJ)**	**Propagationdelay (ns)**	**Supply voltage (V)**	**Classifier**	**Task**	**Accuracy**
Zhong et al. (2021)	50	0.006	N/A	3.3	ANN	Spoken Signal processing	97.6%
Moon et al. (2019)	17.7 × 10^6^ a	54.6	N/A	< 2	CNN	Time-series forecasting	99.2%
Nowshin et al. (2024)	10.8	N/A	500	1.8	RNN-RC	Image recognition	MNIST: 98%
This work	137 b	0.69	280.19	3.3	SNN/DNN	Event representation	DVSGesture: 88.42% NMNIST: 98.15 DVSCIFAR10: 72.45%

^a^Power evaluation for the entire system.

^b^Power evaluation with a single reservoir node.

## 4 Conclusion

In this study, we proposed NEIR, a neuromorphic approach using memristors as reservoir encoders. We utilize the stochastic and non-linear properties of memristors as reservoir node for effective, low-cost feature extraction of event data. Our design does not require a DAC component and directly encode ON and OFF events triggered by digital clock. We also present a simplified memristor model based on the VTEAM model to accurately capture pulse behaviors with fewer parameters. Comparative analyses across four datasets demonstrate that our approach achieves superior accuracy over other methods, illustrating the potential of memristor-based systems in real-time data processing and neuromorphic computing.

## Data Availability

The raw data supporting the conclusions of this article will be made available by the authors, without undue reservation.

## References

[B1] Amir A. Taba B. Berg D. Melano T. McKinstry J. Di Nolfo C.. (2017). “A low power, fully event-based gesture recognition system,” in Proceedings of the IEEE Conference on Computer Vision and Pattern Recognition, 7243–7252. 10.1109/CVPR.2017.78132903824

[B2] Biolek Z. Biolek D. Biolkova V. (2009). Spice model of memristor with nonlinear dopant drift. Radio Eng. 18, 210–214.34955716

[B3] Cao J. Zhang X. Cheng H. Qiu J. Liu X. Wang M.. (2022). Emerging dynamic memristors for neuromorphic reservoir computing. Nanoscale 14, 289–298. 10.1039/D1NR06680C 34932057

[B4] Chen L. Wang X. Yang C. Chen Z. Zhang J. Zeng Z. (2024). Full-analog reservoir computing circuit based on memristor with a hybrid wide-deep architecture. IEEE Trans. Circuits Syst. I: Regul. Pap. 71, 501–514. 10.1109/TCSI.2023.3334267

[B5] Cucchi M. Abreu S. Ciccone G. Brunner D. Kleemann H. (2022). Hands-on reservoir computing: a tutorial for practical implementation. Neuromorphic Comput. Eng. 2:032002. 10.1088/2634-4386/ac7db7

[B6] Delbruck T. Lang M. (2013). Robotic goalie with 3 ms reaction time at 4% cpu load using event-based dynamic vision sensor. Front. Neurosci. 7:69513. 10.3389/fnins.2013.00223 24311999 PMC3836084

[B7] Everding L. Conradt J. (2018). Low-latency line tracking using event-based dynamic vision sensors. Front. Neurorobot. 12:4. 10.3389/fnbot.2018.00004 29515386 PMC5825909

[B8] Furmonas J. Liobe J. Barzdenas V. (2022). Analytical review of event-based camera depth estimation methods and systems. Sensors 22:1201. 10.3390/s22031201 35161946 PMC8838470

[B9] Gallego G. Delbrück T. Orchard G. Bartolozzi C. Taba B. Censi A.. (2020). Event-based vision: a survey. IEEE Trans. Pattern Anal. Mach. Intell. 44, 154–180. 10.1109/TPAMI.2020.3008413 32750812

[B10] Gehrig D. Loquercio A. Derpanis K. G. Scaramuzza D. (2019). “End-to-end learning of representations for asynchronous event-based data,” in Proceedings of the IEEE/CVF International Conference on Computer Vision, 5633–5643. 10.1109/ICCV.2019.00573

[B11] Glover A. Bartolozzi C. (2016). “Event-driven ball detection and gaze fixation in clutter,” in 2016 IEEE/RSJ International Conference on Intelligent Robots and Systems (IROS) (IEEE), 2203–2208. 10.1109/IROS.2016.7759345

[B12] Hu L. Yang J. Wang J. Cheng P. Chua L. O. Zhuge F. (2021). All-optically controlled memristor for optoelectronic neuromorphic computing. Adv. Funct. Mater. 31:2005582. 10.1002/adfm.202005582

[B13] Ielmini D. Wong H.-S. P. (2018). In-memory computing with resistive switching devices. Nat. Electron. 1, 333–343. 10.1038/s41928-018-0092-2

[B14] Jo S. H. Chang T. Ebong I. Bhadviya B. B. Mazumder P. Lu W. (2010). Nanoscale memristor device as synapse in neuromorphic systems. Nano Lett. 10, 1297–1301. 10.1021/nl904092h 20192230

[B15] Khalid M. (2019). Review on various memristor models, characteristics, potential applications, and future works. Trans. Electr. Electron. Mater. 20, 289–298. 10.1007/s42341-019-00116-8

[B16] Kumar D. Li H. Das U. K. Syed A. M. El-Atab N. (2023). Flexible solution-processable black-phosphorus-based optoelectronic memristive synapses for neuromorphic computing and artificial visual perception applications. Adv. Mater. 35:2300446. 10.1002/adma.202300446 37192130

[B17] Kvatinsky S. Ramadan M. Friedman E. G. Kolodny A. (2015). Vteam: a general model for voltage-controlled memristors. IEEE Trans. Circuits Syst. II: Express Br. 62, 786–790. 10.1109/TCSII.2015.2433536

[B18] Lakshmi A. Chakraborty A. Thakur C. S. (2019). Neuromorphic vision: from sensors to event-based algorithms. Data Min. Knowl. Discov. 9:e1310. 10.1002/widm.1310

[B19] Leñero-Bardallo J. A. Carmona-Galán R. Rodríguez-Vázquez A. (2018). Applications of event-based image sensors-review and analysis. Int. J. Circuit Theory Appl. 46, 1620–1630. 10.1002/cta.2546

[B20] Li H. Liu H. Ji X. Li G. Shi L. (2017). Cifar10-dvs: an event-stream dataset for object classification. Front. Neurosci. 11:309. 10.3389/fnins.2017.00309 28611582 PMC5447775

[B21] Liang X. Tang J. Zhong Y. Gao B. Qian H. Wu H. (2024). Physical reservoir computing with emerging electronics. Nat. Electron. 7, 193–206. 10.1038/s41928-024-01133-z

[B22] Lichtsteiner P. Posch C. Delbruck T. (2008). A 128 × 128 120 db 15 μs latency asynchronous temporal contrast vision sensor. IEEE J. Solid-State Circuits 43, 566–576. 10.1109/JSSC.2007.914337

[B23] Lu A. Peng X. Li W. Jiang H. Yu S. (2021). “Neurosim validation with 40nm rram compute-in-memory macro,” in 2021 IEEE 3rd International Conference on Artificial Intelligence Circuits and Systems (AICAS) (IEEE), 1–4. 10.1109/AICAS51828.2021.9458501

[B24] Moon J. Ma W. Shin J. H. Cai F. Du C. Lee S. H.. (2019). Temporal data classification and forecasting using a memristor-based reservoir computing system. Nat. Electron. 2, 480–487. 10.1038/s41928-019-0313-3

[B25] Nowshin F. Huang Y. Sarkar M. R. Xia Q. Yi Y. (2024). Merrc: a memristor-enabled reconfigurable low-power reservoir computing architecture at the edge. IEEE Trans. Circuits Syst. I: Regul. Pap. 71, 174–186. 10.1109/TCSI.2023.3329337

[B26] Orchard G. Jayawant A. Cohen G. K. Thakor N. (2015). Converting static image datasets to spiking neuromorphic datasets using saccades. Front. Neurosci. 9:437. 10.3389/fnins.2015.00437 26635513 PMC4644806

[B27] Pereira M. E. Martins R. Fortunato E. Barquinha P. Kiazadeh A. (2023). Recent progress in optoelectronic memristors for neuromorphic and in-memory computation. Neuromor. Comput. Eng. 3:022002. 10.1088/2634-4386/acd4e2

[B28] Sironi A. Brambilla M. Bourdis N. Lagorce X. Benosman R. (2018). “Hats: histograms of averaged time surfaces for robust event-based object classification,” in Proceedings of the IEEE Conference on Computer Vision and Pattern Recognition, 1731–1740. 10.1109/CVPR.2018.00186

[B29] Soni K. Sahoo S. (2022). “A review on different memristor modeling and applications,” in 2022 International Mobile and Embedded Technology Conference (MECON) (IEEE), 688–695. 10.1109/MECON53876.2022.9752214

[B30] Sun L. Wang Z. Jiang J. Kim Y. Joo B. Zheng S.. (2021). In-sensor reservoir computing for language learning via two-dimensional memristors. Sci. Adv. 7:eabg1455. 10.1126/sciadv.abg1455 33990331 PMC8121431

[B31] Tan G. Wang Y. Han H. Cao Y. Wu F. Zha Z.-J. (2022). “Multi-grained spatio-temporal features perceived network for event-based lip-reading,” in Proceedings of the IEEE/CVF Conference on Computer Vision and Pattern Recognition, 20094–20103. 10.1109/CVPR52688.2022.01946

[B32] Tanaka G. Yamane T. Héroux J. B. Nakane R. Kanazawa N. Takeda S.. (2019). Recent advances in physical reservoir computing: a review. Neural Netw. 115, 100–123. 10.1016/j.neunet.2019.03.005 30981085

[B33] Wu X. Wang S. Huang W. Dong Y. Wang Z. Huang W. (2023). Wearable in-sensor reservoir computing using optoelectronic polymers with through-space charge-transport characteristics for multi-task learning. Nat. Commun. 14:468. 10.1038/s41467-023-36205-9 36709349 PMC9884246

[B34] Wu Y. Deng L. Li G. Zhu J. Xie Y. Shi L. (2019). “Direct training for spiking neural networks: Faster, larger, better,” in Proceedings of the AAAI Conference on Artificial Intelligence, 1311–1318. 10.1609/aaai.v33i01.33011311

[B35] Yan M. Huang C. Bienstman P. Tino P. Lin W. Sun J. (2024). Emerging opportunities and challenges for the future of reservoir computing. Nat. Commun. 15:2056. 10.1038/s41467-024-45187-1 38448438 PMC10917819

[B36] Yang X. You M. Pang L. Du B. (2024). “Reservoir computing based on memristor arrays in random states. IEEE Trans. Circuits Syst. I: Regul. Pap. 71, 3256–3268. 10.1109/TCSI.2024.3394169

[B37] Zhang G. Qin J. Zhang Y. Gong G. Xiong Z.-Y. Ma X.. (2023). Functional materials for memristor-based reservoir computing: dynamics and applications. Adv. Funct. Mater. 33:2302929. 10.1002/adfm.202302929

[B38] Zhong Y. Tang J. Li X. Gao B. Qian H. Wu H. (2021). Dynamic memristor-based reservoir computing for high-efficiency temporal signal processing. Nat. Commun. 12:408. 10.1038/s41467-020-20692-1 33462233 PMC7814066

[B39] Zhong Y. Tang J. Li X. Liang X. Liu Z. Li Y.. (2022). A memristor-based analogue reservoir computing system for real-time and power-efficient signal processing. Nat. Electron. 5, 672–681. 10.1038/s41928-022-00838-3

[B40] Zhou Y. Fu J. Chen Z. Zhuge F. Wang Y. Yan J.. (2023). Computational event-driven vision sensors for in-sensor spiking neural networks. Nat. Electron. 6, 870–878. 10.1038/s41928-023-01055-2

